# Downregulation of BST2 Rescues Cochlear Nerve Demyelination in Age‐Related Hearing Loss via Enhancing Schwann Cell Migration

**DOI:** 10.1111/acel.70325

**Published:** 2025-12-14

**Authors:** Mengxiao Liu, Qi Li, Huan Cao, Huan Yin, Jianwang Yang, Tao Liu, Jiantao Wang, Lei Zhao, Baoshan Wang

**Affiliations:** ^1^ Department of Otolaryngology‐Head and Neck Surgery The Second Hospital of Hebei Medical University Shijiazhuang China

**Keywords:** age‐related hearing loss, bone marrow stromal antigen 2, cell migration, demyelination, POU class 6 homeobox 1, Schwann cells

## Abstract

Cochlear nerve demyelination is a significant pathogenic factor of age‐related hearing loss (ARHL), and Schwann cell (SC) migration function plays a key role in the maintenance and regeneration of myelin sheaths. Here, we found that bone marrow stromal antigen 2 (BST2) is significantly upregulated in cochlear SCs during aging following demyelination and hearing loss. However, specific knockdown of BST2 in SCs could obviously improve the SCs migration and the myelin sheath structure manifested in a reduction of E‐cadherin expression and increased N‐cadherin expression. Further mechanism analysis revealed that POU class 6 homeobox 1 (POU6F1) expression via the NF‐κB pathwayleads to enhanced SCs migration ability and increased expression of the myelin protein zero (MPZ), thereby alleviating nerve demyelination and rescuing hearing loss. This study identifies BST2 as a novel therapeutic target for ARHL intervention. In conclusion, the specific downregulation of BST2 in cochlear SCs rescues age‐related demyelination and hearing loss by activating the POU6F1/NF‐κB pathway, thereby enhancing SC migration capacity and promoting MPZ expression.

## Graphical abstract

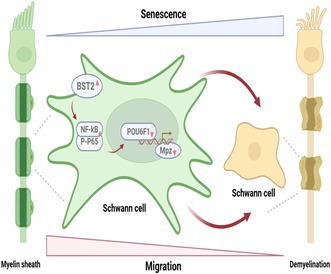

## 1 Introduction

Age‐related hearing loss (ARHL), also known as presbycusis, affects approximately one‐third of individuals over 65 years globally (Rutherford et al. 2018; GBD 2019 Hearing Loss Collaborators 2021), and significantly impairs communication and increases risks of depression and dementia (Li, Ye, et al. 2024; Li, Shao, et al. 2024; Li et al. 2025). In addition, emerging evidence has implicated the significance of auditory neural degeneration in ARHL pathogenesis, in which myelin integrity plays a crucial role in neuronal function (Poleg et al. 2024; Qiu et al. 2024). Nevertheless, the precise mechanism remains incompletely defined.

In the peripheral nervous system (PNS), myelination of spiral ganglion neuron (SGN) axons is exclusively mediated by inner Schwann cells (SCs), which form compact myelin sheaths essential for saltatory conduction and axonal support (Gambarotto et al. 2025). Once injured, SCs undergo dedifferentiation, proliferation, and migration to facilitate axonal re‐engagement and remyelination, and recovery function, in which the migratory function is indispensable for directional translocation to injury sites, establishing contact with regenerating axons, and guiding remyelination (Rogujski et al. 2024; Zerad et al. 2024; Alhamdi et al. 2025). Notably, as a pioneering event in repair processes, impaired migration directly disrupts the initiation of regenerative signaling, reduces targeted delivery of neurotrophic factors, and triggers neuroinflammation, ultimately culminating in demyelination (Park et al. 2019, 2020; Schumacher et al. 2025). More importantly, demyelination not only impairs the signal conduction velocity but also disrupts neurotrophic support, ultimately driving axonal damage and even neuronal death, which further exacerbates hearing loss (Chen et al. 2025; Hsueh et al. 2025; Kong et al. 2025; Schumacher et al. 2025). However, the molecular mechanisms underlying age‐related decline in SC migratory capacity remain poorly understood, severely limiting the development of targeted therapeutic strategies for demyelinating pathologies in ARHL.

Bone marrow stromal antigen 2 (BST2), a type II transmembrane glycoprotein, plays critical roles in diverse physiological and pathological processes. Beyond its well‐established functions in immune regulation, antiviral responses, and tumor development, BST2 participates in fundamental cellular processes including proliferation, migration, adhesion, and apoptosis (Li et al. 2016; Shan et al. 2023; Braun et al. 2024; Marougka et al. 2024; Wen et al. 2024; Zheng et al. 2024). Notably, BST2 is associated with neurodegenerative diseases. In Alzheimer's disease (AD), its expression is significantly upregulated in the hippocampus and frontal cortex, contributing to pathogenesis by disrupting amyloid‐β (Aβ) metabolism and exacerbating neuroinflammation through immune cell activation (Li, Ye, et al. 2024; Li, Shao, et al. 2024). Elevated BST2 expression also occurs in amyotrophic lateral sclerosis (ALS) (Manouchehri et al. 2021). Furthermore, BST2 expression is also upregulated in demyelinating diseases such as multiple sclerosis (MS) and experimental autoimmune encephalomyelitis (EAE) (Manouchehri et al. 2021; Xu et al. 2021). Particularly, in EAE mouse models, specific knockout of BST2 in SCs delays the progression of demyelinating disease (Manouchehri et al. 2021). However, to date, the expression profile and functional significance of BST2 in the cochleae during ARHL, particularly its potential direct relationship with SCs injury and demyelination pathogenesis, as well as the underlying molecular mechanisms, remain unclear in peer‐reviewed literature.

So, in this study, we revealed that BST2 expression in cochlear SCs increases with age. In particular, the decline in SCs' migratory function mediated by BST2 exhibits a positive correlation with hearing impairment, which elucidates a novel mechanism underlying ARHL and identifies a potential therapeutic target for this condition.

## 2 Methods and Materials

### 2.1 Animals and Groups

All the animal experiments were approved by the Animal Care and Ethics Committee of the Second Hospital of Hebei Medical University (No. 2022‐AE308). The male C57BL/6 mice were purchased from Charles River Labs and housed under a 12 h light/dark cycle at a constant temperature of 22°C ± 1°C and relative humidity of 55%–60%, with free access to food and water. One week later, the mice were randomly grouped as follows: (1) To investigate age‐dependent changes in BST2 expression in mice cochleae, mice aged 3, 6, 9, 12, 15, and 18 months were subjected to immunofluorescence staining of cochlear sections. (2) To investigate the role of BST2 in the cochlear SCs damage, the 2‐month‐old and 6‐month‐old mice were assigned to the 4M‐NC1 group (AAV9‐MBP‐EGFP, *n* = 6) and 8M‐NC1 group (AAV9‐MBP‐EGFP, *n* = 7), respectively, along with the 8M‐shBST2 group (AAV9‐MBP‐shBST2‐EGFP, *n* = 6). (3) Additionally, to examine the effects of POU6F1 on cochlear SCs damage, the 6‐month‐old mice were divided into the 8M‐NC2 group (AAV9‐MBP‐mCherry, *n* = 10) and 8M‐oePOU6F1 group (AAV9‐MBP‐POU6F1‐mCherry, *n* = 10). The corresponding viruses were injected into the posterior semicircular canal of the mice using the methods and dosages described previously (Yin et al. 2025). (4) Furthermore, to verify the impact of the interaction between BST2 and POU6F1 on the migratory function of inner ear SCs, the 6‐month‐old mice were further categorized into the NC1 + NC2 group (AAV9‐MBP‐EGFP + AAV9‐MBP‐mCherry, *n* = 10), shBST2+NC2 group (AAV9‐MBP‐shBST2‐EGFP + AAV9‐MBP‐mCherry, *n* = 10), and shBST2+shPOU6F1 group (AAV9‐MBP‐shBST2‐EGFP + AAV9‐MBP‐shPOU6F1‐mCherry, *n* = 10). For co‐injections, 1 μL of each corresponding virus was thoroughly mixed and then injected (Omichi et al. 2020). All mice were sacrificed after 2 months.

### 2.2 Immunofluorescence (IF)

The detailed methods for cochlear sample preparation, sectioning, and fluorescent staining were described previously (Yin et al. 2025). The antibodies used are as follows: anti‐MBP (1:200, Biolegend, #808403), anti‐TUJ1 (1:200, Biolegend, #801202), anti‐Ki67 (1:50, Servicebio, #GB111499), anti‐SOX10 (1:400, Proteintech, #66786–1‐Ig), anti‐E‐cadherin (1:50, Proteintech, #20874‐1‐AP), anti‐N‐cadherin (1:50, Proteintech, #22018‐1‐AP), anti‐MAG (1:500, HUABIO, #HA721818), anti‐SOX2 (1:500, HUABIO, #HA721155), anti‐BST2 (1:50, Santa Cruz, # sc‐390719), anti‐POU6F1 (1:50, Abclonal, #A7299), and anti‐MPZ (1:50, Proteintech, #10572‐1‐AP). Images were captured using a fluorescence microscope (Zeiss 900 confocal system).

### 2.3 Transmission Electron Microscopy (TEM)

C57BL/6J mice were anesthetized and rapidly perfused with electron microscopy fixative (2.5% glutaraldehyde and 4% paraformaldehyde). Next, the cochleae were harvested by decapitation and then fixed in the same fixative at 4°C in the dark for 2 h. Ultrathin sections were prepared through decalcification, ethanol dehydration, epoxy resin embedding, and staining with saturated uranyl acetate in alcohol and lead citrate solutions. Then, the myelin structures were observed and imaged using a HT7800 Transmission Electron Microscope (TEM) (Hitachi, Japan) at magnifications of 1.2 k× and 40 k×.

### 2.4 Luxol Fast Blue (LFB) Staining

According to the manufacturer's protocol (Solarbio, China), frozen cochlear sections were stained with LFB solution, differentiated using a differentiation solution, and counterstained with eosin. After mounting, microscopic images were acquired, and the stained regions were quantitatively analyzed using Image J software.

### 2.5 Immunohistochemistry (IHC)

Immunohistochemical staining followed the previously described method (Yin et al. 2025). The primary antibody used was anti‐MBP (1:200, Santa Cruz, #sc‐271524) and was imaged under a light microscope (Olympus, Japan).

### 2.6 Oil Red O Staining

The cochlear frozen sections were washed in PBS and incubated in 60% Oil Red O solution, pre‐filtered through a 0.2 μm filter, for 15 min at room temperature. The stained sections were rinsed with 60% isopropanol and PBS to reduce background interference. Randomly selected images were captured using an Olympus microscope (BX71, Japan).

### 2.7 Auditory Brainstem Response (ABR)

ABR measurements were carried out as previously described (Lu et al. 2024). The latencies of ABR wave I were specifically measured in response to a 90 dB click stimulus. The amplitude is calculated as the distance from the peak to the baseline.

### 2.8 TUNEL Staining

Apoptotic cells were detected using the terminal deoxynucleotidyl transferase (TdT)‐mediated dUTP nick end labeling (TUNEL) assay according to the manufacturer's instructions (Vazyme, China). Fluorescent images were captured using a confocal fluorescence microscope (Leica TCS SPE).

### 2.9 Single‐Cell Sequencing

Single‐cell sequencing was conducted by CapitalBio Technology (Beijing, China) following standard procedures. In brief, cochlear tissues from 2‐month‐old and 12‐month‐old mice (15 mice per sample) were processed under a stereomicroscope to remove the bone walls, and cell suspensions were obtained after digestion, incubation, centrifugation, filtration, washing. cDNA was generated via 10× Genomics platform processing, with subsequent library construction, sequencing, data preprocessing, cell clustering, and annotation. Differentially expressed genes were identified (|fold change| > 1.0, *p* < 0.05).

### 2.10 Quantitative Real‐Time PCR (qRT‐PCR)

Total RNA was extracted using TRIzol reagent (Invitrogen, USA). The extracted RNA was then reverse transcribed into cDNA using the HiFiScript cDNA Synthesis Kit (Roche). Subsequently, Q‐PCR was performed according to the manufacturer's protocol (Promega, USA). Gene expression levels were determined using the comparative threshold cycle (2^−ΔΔCt^) method. The primer sequences are listed in Table S1.

### 2.11 Cell Culture and Groups

The rat Schwann cell line (RSC96, ATCC CRL‐2765) was regularly cultured in DMEM (Gibco, USA) supplemented with 10% FBS (Lonsera, Uruguay) in a humidified incubator at 37°C with 5% CO_2_. The cells were grouped as follows: (1) To investigate the effect of doxorubicin (DOX) on the SCs injury and the expression of BST2, the SCs were exposed to DOX solutions at concentrations of 0, 200, 400, and 600 μM, with further refinement to concentrations of 0, 400, 450, 500, 550, and 600 μM. Sample of DOX solution dissolved in DMSO were collected at 0, 12, 24, 48 h, respectively. The MTS assay was used to determine cell viability according to the manufacturer's instructions (Sigma, USA). (2) To further confirm the correlation between BST2/POU6F1 and SCs injury, *Bst2*‐siRNA, *Pou6f1*‐siRNA (Hanbio, China), or plasmids (Hanbio, China) along with their respective controls were transfected using Lipofectamine 2000 (Invitrogen, USA). After 24 h, the culture medium was replaced and DOX was added. Then the cells were harvested for subsequent experiments. (3) To assess whether the AKT/NF‐κB pathways mediated the change of POU6F1 or not, the cells were pretreated with 10 μM LY294002 (Sigma, #440202) and BAY11‐7082 (Sigma, #B5556) for 2 h, then cultured with DOX medium for 24 h. Then the cells were collected and relevant indicators were detected.

### 2.12 β‐Galactosidase Staining

Cellular senescence was evaluated using a β‐galactosidase staining kit (Beyotime, China). Briefly, cells were PBS‐rinsed, fixed with staining fixative, washed thrice, and incubated with 1 mL staining solution overnight at 37°C in a CO_2_‐free incubator.

### 2.13 Cell Migration Assay

Cell migration was assessed using Transwell and scratch assays. For Transwell, RSC96 cells were seeded in upper chambers with 650 μL 10% FBS medium in lower chambers, incubated at 37°C for 48 h, fixed/stained with 1% crystal violet, and quantified in three random fields. For scratch assays, confluent RSC96 monolayers in 6‐well plates were scratched with 10 μL pipette tips, PBS‐washed, cultured in 1% FBS medium, and took photos at 0 h and 48 h (Olympus, Japan).

### 2.14 Western Blot

The experimental method was conducted as previously described (Yin et al. 2025), and the primary antibodies used included: anti‐BST2 (1:200, Santa Cruz, # sc‐390719), anti‐ATP1A1 (1:10000, Proteintech, #14418‐1‐AP), anti‐E‐cadherin (1:20000, Proteintech, #20874‐1‐AP), anti‐ N‐cadherin (1:2000, Proteintech, #22018‐1‐AP), anti‐VIMENTIN (1:2000, Proteintech, #10366‐1‐AP), anti‐β‐actin (1:5000, Proteintech, #66009‐1‐Ig), anti‐POU6F1 (1:200, Abclonal, #A7299), anti‐P‐ERK (1:3000, Proteintech, #28733‐1‐AP), anti‐AKT (1:1000, Proteintech, #10176‐2‐AP), anti‐P‐AKT (1:2000, CST, #4060T), anti‐P65 (1:200, Santa Cruz, #sc‐8008), anti‐P‐P65 (1:1000, Biolegend, #3033T), and anti‐MPZ (1:500, Proteintech, #10572‐1‐AP).

### 2.15 RNA Sequencing (RNA‐Seq)

Cells from the DMSO+NC group, DOX+NC group, and DOX+siBST2 group preserved in Trizol (Invitrogen, USA) were sent to Majorbio (Shanghai, China) for RNA purification, reverse transcription, library construction, and sequencing. Differentially expressed genes were identified through comparative analysis among these groups (|fold change| > 2.0, *p* < 0.05), and volcano plots were generated by the DESeq2 software.

### 2.16 Chromatin Immunoprecipitation (ChIP)

According to the manufacturer's instructions, the ChIP assay was performed using the Pure Binding Chromatin Immunoprecipitation Kit (GENESEED, China). Samples were sonicated and immunoprecipitated using IgG or a flag tag antibody (4 μg/mL, Proteintech, #20543‐1‐AP). The recovered DNA was used as a template for qRT‐PCR analysis. The primers are listed in Table S1.

### 2.17 Statistical Analyses

Data were analyzed using GraphPad Prism 9 and were expressed as the mean ± standard deviation. Independent sample *t*‐tests or nonparametric tests were performed as appropriate. The Spearman test was conducted to evaluate the relationship between the two datasets. *p* < 0.05 was considered statistically significant.

## 3 Results

### 3.1 Impaired Schwann Cell Migration Plays a Critical Role in Demyelination of ARHL

**FIGURE 1:**
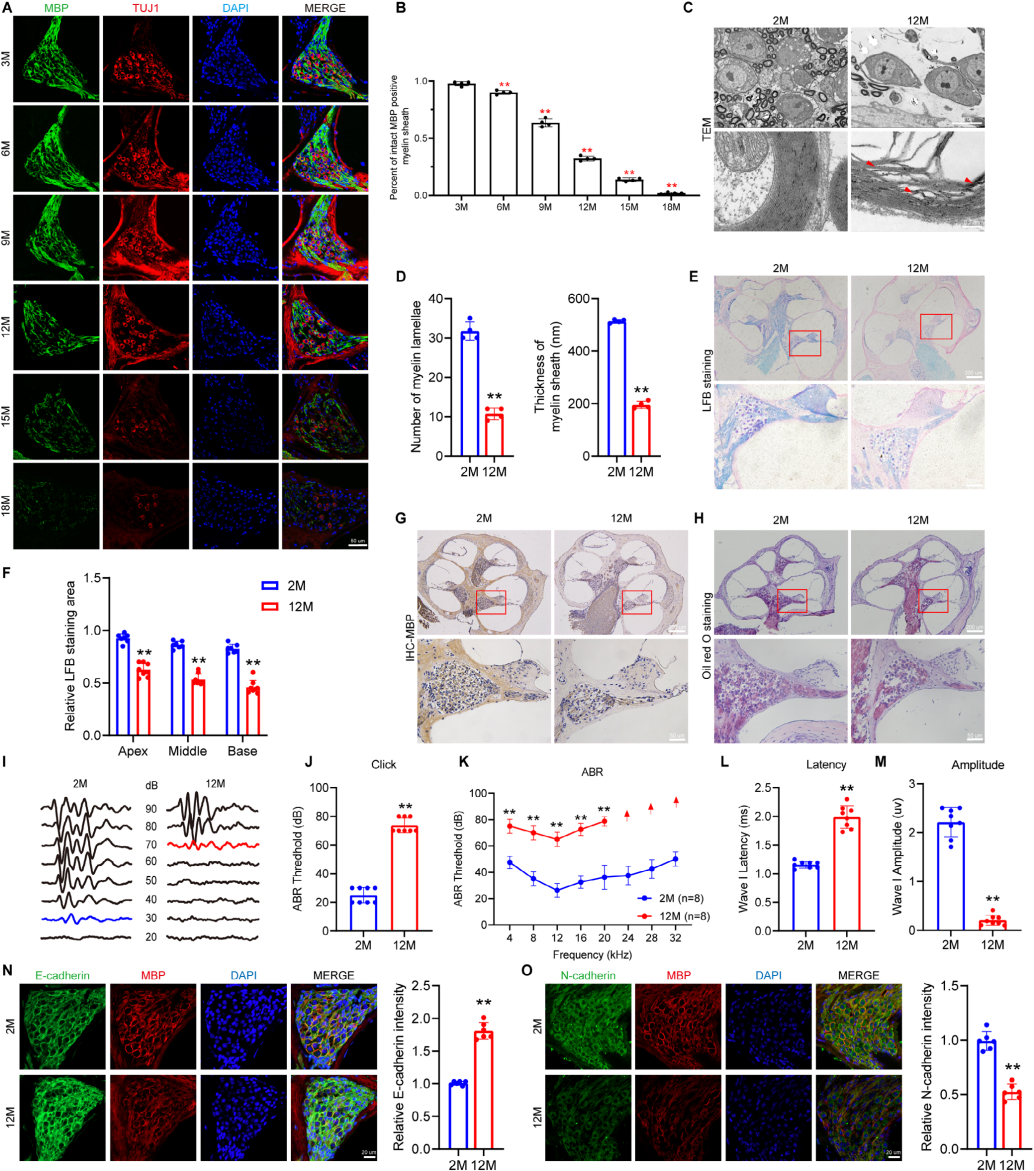
Demyelination in the cochlea of mice with ARHL. (A) MBP (green, SCs) and TUJ1 (red, neurons) in SGNs from WT 3M, 6M, 9M, 12M, 15M, and 18M C57BL/6J mice by immunofluorescence. (B) Quantification of SGNs with intact MBP^+^ myelin sheaths. (C) TEM examination of the cochlea from 2M and 12M C57BL/6J mice. (D) Quantitative analysis of myelin lamellae and myelin sheath thickness. (E) LFB staining showed the distribution of myelin in the cochlea of WT 2M and 12M C57BL/6J mice. (F) Quantitative analysis of LFB staining at SGN areas. (G) IHC was used for detecting MBP expression in the cochlea of 2M and 12M C57BL/6J mice. (H) Oil red O staining showed the lipid distribution in the cochlea of WT 2M and 12M C57BL/6J mice. (I) Representative ABR waveforms in response to clicking (90–20 dB SPL) sound pressure levels in WT 2M and 12M C57BL/6J mice. (J, K) Click values and ABR thresholds to pure‐tones were measured in WT 2M and 12M C57BL/6J mice. (L‐M) Amplitude and latency of ABR wave I were measured with click at SPL of 90 dB in WT 2M and 12M C57BL/6J mice. Immunofluorescence showed expression of E‐cadherin (N) and N‐cadherin (O) (green) in the SGNs of the cochleae in WT 2M and 12M mice. SCs were labeled with MBP (red). Nuclei were stained with DAPI (blue). Data are presented as means ± SEM. *p < 0.05, **p < 0.01 versus 2M.

First, as shown in Figure 1A,B, there were an age‐related reduction in intact MBP^+^ myelin sheaths in SGNs from 3, 6, 9, 12, 15, and 18 months old (Figure 1A,B). More notably, the decline began at 6 months and became significant at 12 months. Similarly, TEM revealed myelin lamellar disintegration in cochlear neurons of 12‐month‐old mice, characterized by the loss of dense structure and reduced lamellar thickness (Figure 1C,D). Additionally, LFB staining revealed reduced myelin staining areas in the apical, middle, and basal turns of the cochleae in 12‐month‐old mice compared with 2‐month‐old mice (Figure 1E,F). IHC further confirmed the weaker MBP expression in 12‐month cochleae (Figure 1G). In addition, Oil Red O staining indicated a significant decrease in sphingomyelin content in the spiral ganglion and osseous spiral lamina regions in 12‐month‐old mice (Figure 1H), further confirming myelin structure damage in neurons during ARHL.

Furthermore, the auditory function measurements showed that compared to 2‐month‐old C57BL/6J mice, 12‐month‐old mice exhibited a significant increase in click auditory thresholds (Figure 1I,J) and tone thresholds across all frequencies (*p* < 0.01, Figure 1K), while the ABR wave I latency was significantly prolonged (Figure 1L) and the amplitude decreased (Figure 1M) in 12‐month‐old mice. Concomitantly, SCs' migratory function declined, evidenced by decreased N‐cadherin and increased E‐cadherin expression (*p* < 0.01, Figure 1N,O), indicating age‐related impairment of cochlear SCs' motility.

### 3.2 Elevated BST2 Expression Mediates the Dysfunction of Schwann Cell Migration in ARHL

**FIGURE 2:**
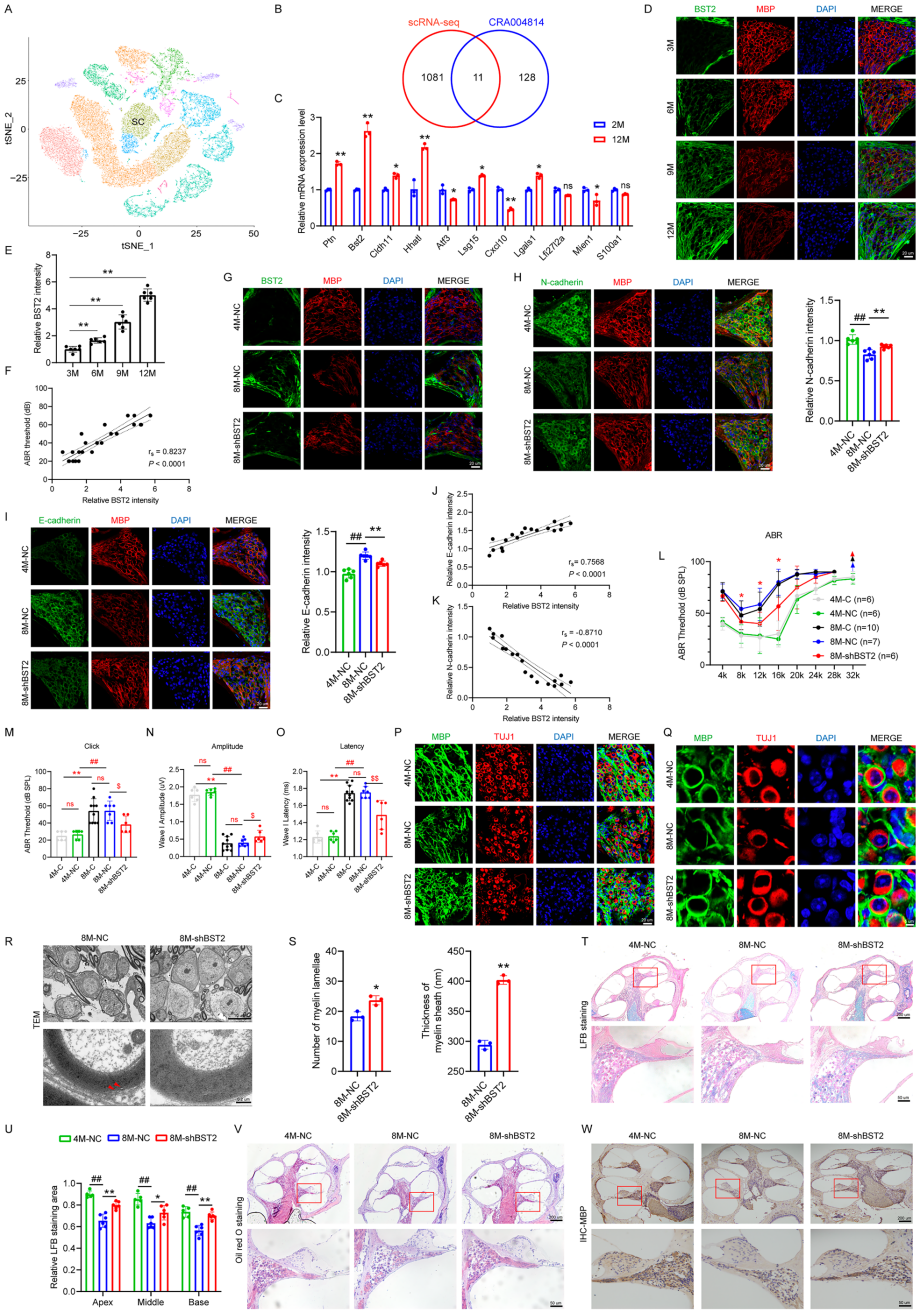
Downregulating BST2 can improve SCs migration capacity and alleviate neural demyelination in ARHL. (A) tSNE plot showing the distribution of different cell types in the cochlea. (B) The Venn diagram showed the intersection result of scRNA‐seq and CRA004814, and there was a total of 11 genes. (C) The mRNA levels of the 11 genes in the cochlea of 2M and 12M C57BL/6J mice measured by qRT‐PCR assay. (D‐E) Expression of BST2 (green) in the cochlea of 3M, 6M, 9M, and 12M C57BL/6J mice by immunofluorescence. SCs were labeled with MBP (red). Nuclei were stained with DAPI (blue). (F) Correlation analysis of BST2 expression and ABR thresholds. (G) Expression of BST2 (green) and MBP (red) in the SGNs of the cochlea in the 4M‐NC, 8M‐NC, and 8M‐shBST2 groups. Immunofluorescence showed expression of N‐cadherin (H) and E‐cadherin (I) (green) in the cochlea of 4M‐NC, 8M‐NC, and 8M‐shBST2 groups. SCs were labeled with anti‐MBP (red). (J, K) Correlation analysis of N‐cadherin and E‐cadherin expression with BST2 expression. (L, M) ABR threshold to pure tones at tested frequencies and click values were measured. (N, O) Latency and amplitude of ABR wave I were measured with click at SPL of 90 dB. (P) Expression of MBP (green) in SGNs of the 4M‐NC, 8M‐NC, and 8M‐shBST2 groups by immunofluorescence. SGNs were labeled with anti‐TUJ1 (red). (Q) Enlarged images of neurons in the cochlea in the 4M‐NC, 8M‐NC, and 8M‐shBST2 groups. Nuclei were stained with DAPI (blue). (R) TEM images of the cochlea from 8M‐NC and 8M‐shBST2 groups. (S) Quantitative analysis of myelin lamellae and myelin sheath thickness. (T) LFB staining showed the distribution of myelin in the cochlea of 4M‐NC, 8M‐NC, and 8M‐shBST2 groups. (U) Quantitative analysis of LFB staining at SGN areas. (V) Oil Red O staining showed the lipid distribution in the cochlea of 4M‐NC, 8M‐NC, and 8M‐shBST2 groups. (W) IHC was used for detecting MBP expression in the cochlea of 4M‐NC, 8M‐NC, and 8M‐shBST2 groups. Data are presented as means ± SEM. *p < 0.05, **p < 0.01; #*p* < 0.05, ##*p* < 0.01.

To further explore the molecular mechanisms underlying neural demyelination and Schwann cell migration dysfunction in ARHL mice, we performed single‐cell transcriptome sequencing on cochlear tissues from 2‐month‐old (the young group) and 12‐month‐old (the aged group) C57BL/6J mice. Cells were classified into 14 major types (Figure 2A). Focusing on the Schwann cell population, we integrated differentially expressed genes with a public dataset (CRA004814) (Sun et al. 2023) and successfully identified 11 differentially expressed genes (Figure 2B). qRT‐PCR validation using total cochlear RNA from mice revealed that *Bst2* mRNA levels significantly increased in 12‐month‐old mice compared to 2‐month‐old (*p* < 0.01, Figure 2C). Additionally, IF showed that positive signals of BST2 protein were primarily localized in the cell membranes of cochlear SCs, and the expression increased in an age‐dependent manner (Figure 2D). Further double labeling of Schwann cells with MBP and BST2 revealed significantly elevated BST2 expression in aged mouse cochlear SCs (Figure 2E), which positively correlated with auditory thresholds (Figure 2F).

To assess BST2's effect on SCs function in ARHL mice, we used posterior semicircular canal microinjection to deliver AAV into the cochlea of 2‐ and 6‐month‐old mice. IF revealed significantly lower BST2 expression in SGNs areas of the 8M‐shBST2 group versus the 8M‐NC group, confirming successful AAV delivery and BST2 knockdown (Figure 2G). Further evaluation of BST2's impact on SCs migration revealed that downregulating BST2 in SCs reduced E‐cadherin expression (*p* < 0.01, Figure 2H) and increased N‐cadherin expression (*p* < 0.05, Figure 2I), correlating with BST2 levels (*p* < 0.01, Figure 2J,K), which suggests that downregulating BST2 could enhance the SCs migration ability.

ABR test revealed no significant differences in hearing between the untreated groups and the groups injected with empty virus, indicating that the surgical procedure had no impact on auditory function (Figure 2L–O). Furthermore, compared to the 4M‐NC group, 8M‐NC mice exhibited elevated ABR thresholds, prolonged wave I latencies, and reduced amplitudes (Figure 2L–O). In contrast, 8M‐shBST2 mice showed decreased click auditory thresholds and reduced pure‐tone ABR thresholds at 8, 12, and 16 kHz (*p* < 0.05, Figure 2L,M), along with significantly shortened wave I latencies and increased amplitudes (Figure 2N,O), suggesting alleviated hearing loss in ARHL mice.

Additionally, compared to the 4M‐NC group, the 8M‐NC group exhibited a reduced number of neurons in the spiral ganglion region, decreased MBP expression, and myelin defects and fractures (Figure 2P,Q). In contrast, the 8M‐shBST2 group showed an increased number of neurons with intact myelin sheaths and more complete myelin morphology compared to the 8M‐NC group (Figure 2P,Q). Furthermore, TEM revealed that the 8M‐shBST2 group had an increased number of neurons, with more numerous and thicker myelin lamellae and more regular myelin structures (Figure 2R,S). Moreover, LFB staining demonstrated that the 8M‐shBST2 group had an expanded staining area in the spiral ganglion regions of the cochlear apical, middle, and basal turns compared to the 8M‐NC group (Figure 2T,U). Oil Red O staining and immunohistochemical analysis indicated that specifically downregulating BST2 in SCs reduced the loss of myelin sphingomyelin and proteins in the cochlea (Figure 2V,W). The above results indicate that downregulating BST2 in SCs can enhance cell migration ability and delay the onset of ARHL.

**FIGURE 3:**
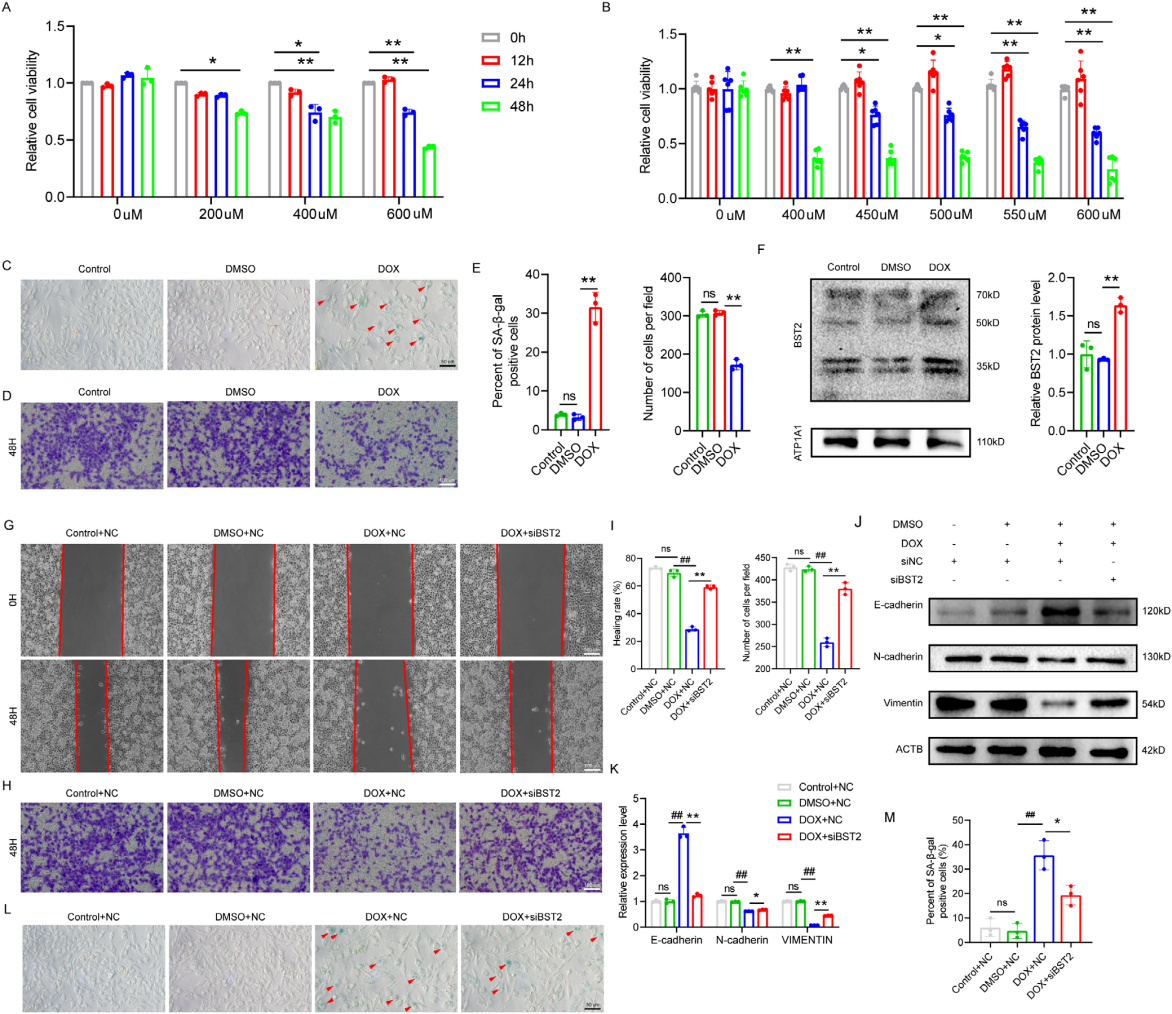
Construction of an RSC96 cell migration impairment model by DOX. (A, B) The effect of DOX concentration and induction duration on cell viability was detected by MTS assay. (C) SA‐β‐gal staining for Schwann cell. The cells marked by red arrows are senescent cells. (D) The cell migration ability of SC was assessed by Transwell assay. (E) Quantitative analysis of SA‐β‐gal staining and Transwell. (F) The effect of DOX on the expression of BST2 in Schwann cell was examined by Western blot. (G–I) Cell migration was measured by Transwell and scratch assay. (J, K) The expression of migration‐related proteins was detected by Western blot. (L, M) SA‐β‐gal staining and quantitative analysis for SCs. The data are expressed as the mean ± SD. *p < 0.05, **p < 0.01; #*p* < 0.05, ##*p* < 0.01.

To further explore the role of BST2 in functional alterations of aging SCs, the Schwann cell line RSC96 was incubated with doxorubicin (DOX) (Eggers et al. 2019; Tang et al. 2025). MTS assays showed that cell viability gradually declined in a time‐ and concentration‐dependent manner (Figure 3A,B). Subsequently, β‐galactosidase staining and Transwell assays revealed a significant increase in the proportion of senescence‐positive cells and a marked reduction in the number of cells migrating to the lower chamber in the DOX group, indicating successful establishment of a senescence model with impaired Schwann cell migration (Figure 3C–E). Moreover, we found that DOX treatment significantly upregulated BST2 expression (*p* < 0.01, Figure 3F).

To further investigate the impact of BST2 on the migration function of aging RSC96 cells, si*Bst2* was transfected into RSC96 cells (Figure S1) and then treated with 550 nM DOX for 24 h. The scratch assay showed that the healing rate in the SCs group with BST2 knockdown was faster than that in the control group (*p* < 0.01, Figure 3G). In addition, as shown in Figure 3H, knockdown of BST2 could significantly increase the SCs migration mediated by DOX. Additionally, western blot revealed that BST2 knockdown upregulated N‐cadherin and VIMENTIN expression while downregulating the E‐cadherin expression (Figure 3J,K). While senescence staining showed a significant reduction in the proportion of senescent cells after BST2 knockdown (*p* < 0.05, Figure 3M). These experimental results prove that knocking down BST2 in aging SCs could improve cell migration function and delay senescence.

### 3.3 BST2 Impairs Schwann Cell Migration by Downregulating POU6F1 to Suppress *Mpz* Transcription

**FIGURE 4:**
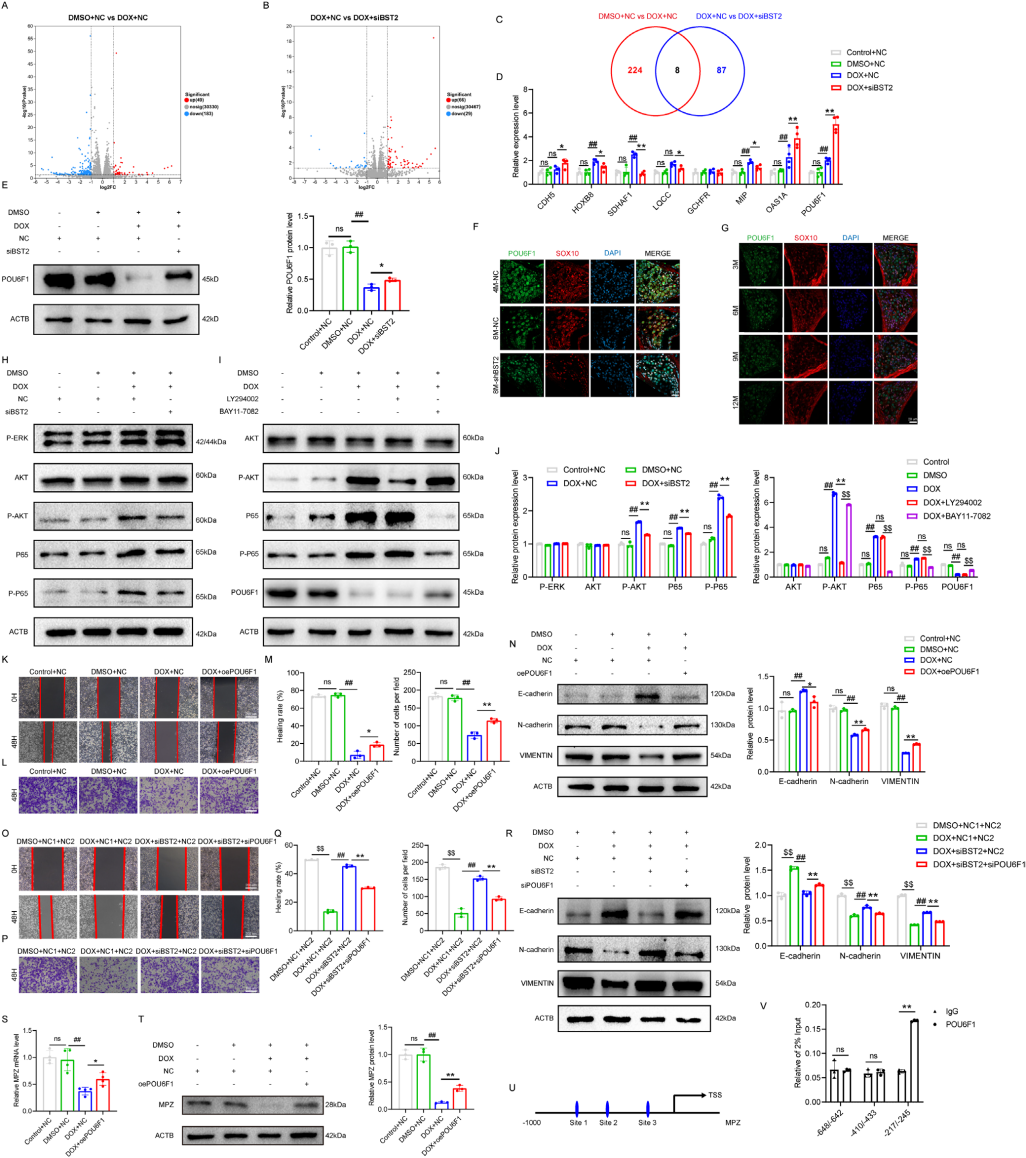
Downregulation of POU6F1 mediated by BST2 compromises Schwann cell migration in ARHL. (A) Volcano plot illustrating the differentially expressed genes between the DOX + NC and DMSO + NC groups. (B) Volcano plot of the differentially expressed genes in DOX + NC and DOX + siBST2 group. Red dots indicate genes with higher expression levels, while blue dots represent genes with lower expression levels. (C) Venn diagram of candidate genes. The total count of numbers within each circle signifies the overall number of differentially expressed mRNAs identified in the pairwise comparison, while the intersecting areas of the circles denote the commonly differentially expressed mRNAs shared among them. (D) The qRT‐PCR assay showed that the expression of candidate downstream genes (E) The Western blot revealed the expression level of POU6F1 protein. (F) Expression of POU6F1 (green) in the cochlea of 4 M‐NC, 8M‐NC, and 8M‐shBST2 mice was measured by immunofluorescence. (G) Expression of POU6F1 (green) on SCs in the cochlea of 3M, 6M, 9M, and 12M C57BL/6J mice by immunofluorescence. SCs were labeled with SOX10 (red). Nuclei were stained with DAPI (blue). (H–J) Western Blot analysis was performed to detect the expression of proteins involved in the signaling pathway. (K–M) The impact of POU6F1 on the migration function of SCs was investigated by Transwell and scratch assay. (N) The Western blot was performed to verify the effect of POU6F1 on the expression of migration‐related proteins. (O–Q) Cell migration was measured by Transwell and scratch assay. (R) The expression of migration‐related proteins was detected by Western blot. (S, T) The results from qRT‐PCR and Western blot demonstrated the changes in MPZ expression levels after overexpression of POU6F1. (U, V) The binding site of the MPZ promoter with POU6F1 was verified by ChIP‐qPCR. Data are presented as means ± SEM. *p < 0.05, **p < 0.01; ^#^ *p* < 0.05, ^##^ *p* < 0.01; ^$^ *p* < 0.05, ^$$^ *p* < 0.01.

As shown in Figure 4A–C, RNA sequencing analysis identified eight common differentially expressed genes (*Cdh5*, *Hoxb8*, *Sdhaf1*, *Lqcc*, *Gchfr*, *Mip*, *Oas1a*, and *Pou6f1*) between the two comparison groups. Further qRT‐PCR verification revealed that POU6F1 was the most markedly altered gene in the BST2 knockdown group (Figure 4D). Both cellular and animal experiments similarly confirmed a marked increase in POU6F1 expression levels after BST2 knockdown (Figure 4E,F). Furthermore, as mice aged, the fluorescence intensity of POU6F1 gradually diminished (Figure 4G).

To deeply elucidate the regulatory mechanism of BST2 on POU6F1, the ERK, AKT, and P65 signal pathways were detected by Western blot. As illustrated in Figure 4H, the DOX treatment significantly increased the expression of P‐AKT, P65, and P‐P65, while BST2 knockdown reduced the expression of these proteins. Further validation using inhibitors showed that the NF‐κB pathway inhibitor BAY 11‐7082 markedly increased POU6F1 expression, whereas the AKT pathway inhibitor LY294002 had no significant effect on POU6F1 expression (Figure 4I,J), suggesting that elevated BST2 might activate the NF‐κB pathway and, in turn, downregulate the POU6F1 expression.

Then, to assess POU6F1's role in SCs migration, we performed plasmid‐mediated overexpression (Figure S1). The scratch healing assay revealed that overexpressing POU6F1 exhibited faster healing of the scratch area compared to the control group (Figure 4K). Similarly, the Transwell assay showed a significant increase in the number of SCs migrating through the Transwell membrane in the overexpression group (Figure 4L). Meanwhile, overexpression of POU6F1 led to increased expression of N‐cadherin and VIMENTIN proteins, while downregulating E‐cadherin protein expression (Figure 4N). Additionally, rescue experiments demonstrated that siPOU6F1 intervention reduced the scratch healing rate and the number of migrating cells in the siBST2 group (Figure S1 and Figure 4O–Q), and attenuated the upregulation of N‐cadherin and VIMENTIN, as well as partially reversed the downregulation of E‐cadherin (Figure 4R). These findings suggest that BST2 downregulates POU6F1 by activating the NF‐κB pathway, thereby mediating the decline in SCs migration function.

Previous studies have revealed that POU6F1, as a transcription factor, can bind to the promoter of MPZ in SCs and regulate its expression (Toda et al. 2008). Our experiments found that in a cellular senescence model, both the mRNA and protein levels of MPZ decreased, while overexpression of POU6F1 increased its expression (Figure 4S,T). Additionally, we designed primers based on the top three predicted binding sites from the JASPAR website, and ChIP‐qPCR demonstrated enrichment of POU6F1 at the −217 to −245 region of the MPZ promoter (Figure 4U,V).

### 3.4 BST2‐Mediated POU6F1 Downregulation Impairs Schwann Cell Migration and Drives Demyelination in ARHL

**FIGURE 5:**
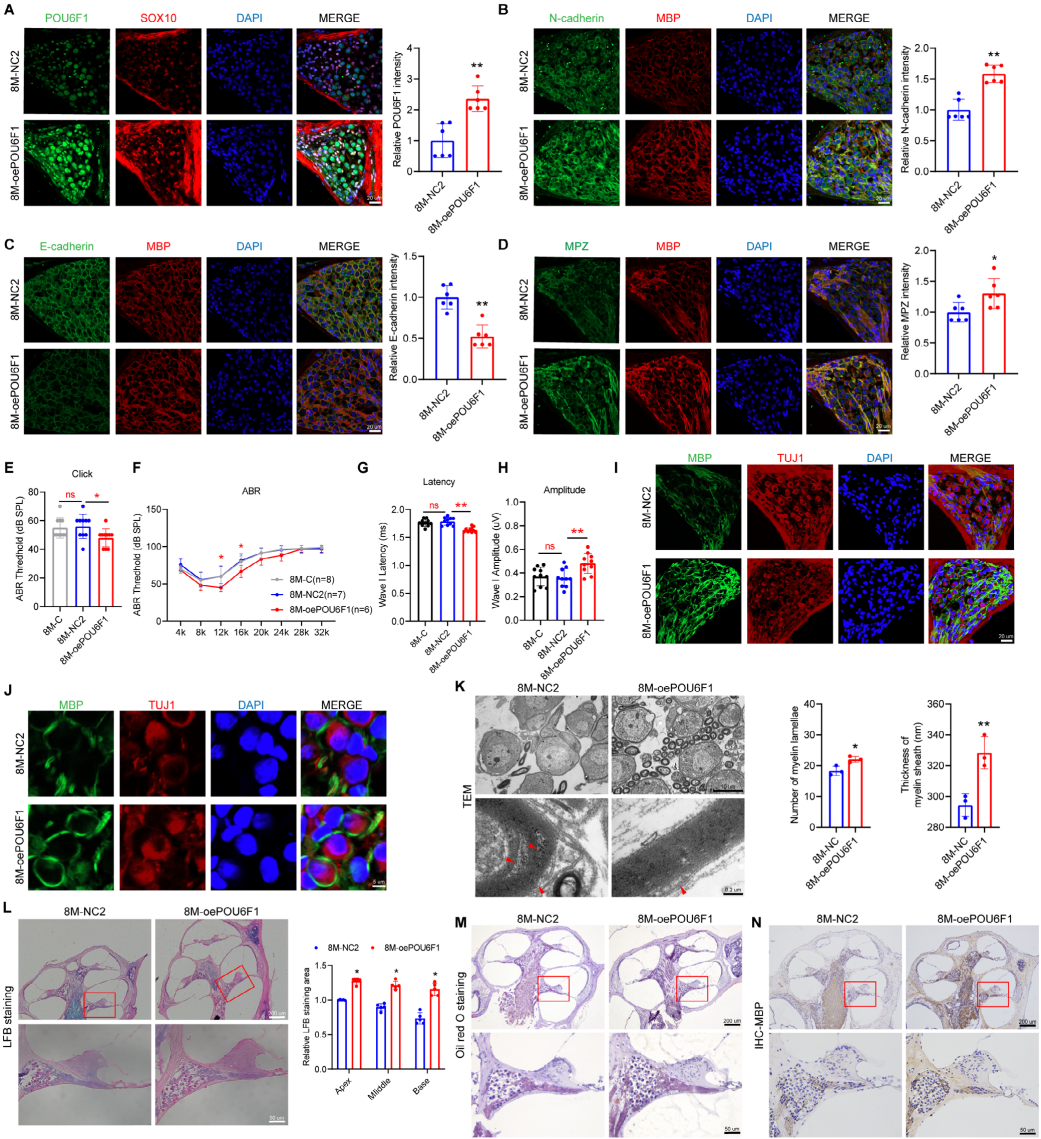
POU6F1 promotes Schwann cell migration and alleviates nerve demyelination. (A) Expression of POU6F1 (green) and SOX2 (red) in the SGNs of the cochlea in 8M‐NC2 and 8M‐oePOU6F1. Immunofluorescence showed expression of N‐cadherin (B), E‐cadherin (C), and MPZ (D) (green) in the cochlea of 8M‐NC2 and 8M‐oePOU6F1. SCs were labeled with anti‐MBP (red). (E, F) Click values and ABR thresholds to pure tones were measured in 8M‐C, 8M‐NC2, and 8M‐oePOU6F1. (G, H) Latency and amplitude of ABR wave I were measured with click at SPL of 90 dB in 8M‐C, 8M‐NC2, and 8M‐oePOU6F1. (I, J) Expression of MBP (green) in SGNs of 8M‐C, 8M‐NC2, and 8M‐oePOU6F1 by immunofluorescence. SGNs were labeled with anti‐TUJ1 (red). Nuclei were stained with DAPI (blue). (K) TEM images of the cochlea from 8M‐C, 8M‐NC2, and 8M‐oePOU6F1. (L) LFB staining showed the distribution of myelin in the cochlea of 8M‐C, 8M‐NC2, and 8M‐oePOU6F1. (M) Oil Red O staining showed the lipid distribution in the cochlea of 8M‐C, 8M‐NC2, and 8M‐oePOU6F1. (N) Immunohistochemistry was used for detecting MBP expression in the cochlea of 8M‐C, 8M‐NC2, and 8M‐oePOU6F1. Data are presented as means ± SEM. *p < 0.05, **p < 0.01 versus 8M‐NC2. ^#^ *p* < 0.05, ^##^ *p* < 0.01.

To further clarify the role of POU6F1 in the migratory function of SCs in the cochlea of ARHL mice, we injected encapsulating POU6F1 AAV into 6‐month‐old mice (Figure 5A). As shown in Figure 5B,C, the expression of N‐cadherin in the cochlea increased while E‐cadherin decreased in the oePOU6F1 group. Concurrently, the expression of MPZ increased (Figure 5D). Additionally, overexpression of POU6F1 reduced the ABR and click thresholds (Figure 5E,F), increased the amplitude of wave I, and decreased its latency in mice (Figure 5G,H), indicating improved hearing in ARHL mice.

Furthermore, the oePOU6F1 group exhibited milder myelin pathology, characterized by an increased proportion of SGNs with intact myelin sheaths, more complete and regular myelin structures (Figure 5I–K), an expanded area of LFB staining (Figure 5L), and reduced loss of myelin phospholipids and proteins in the cochlear myelin sheath (Figure 5M,N).

**FIGURE 6:**
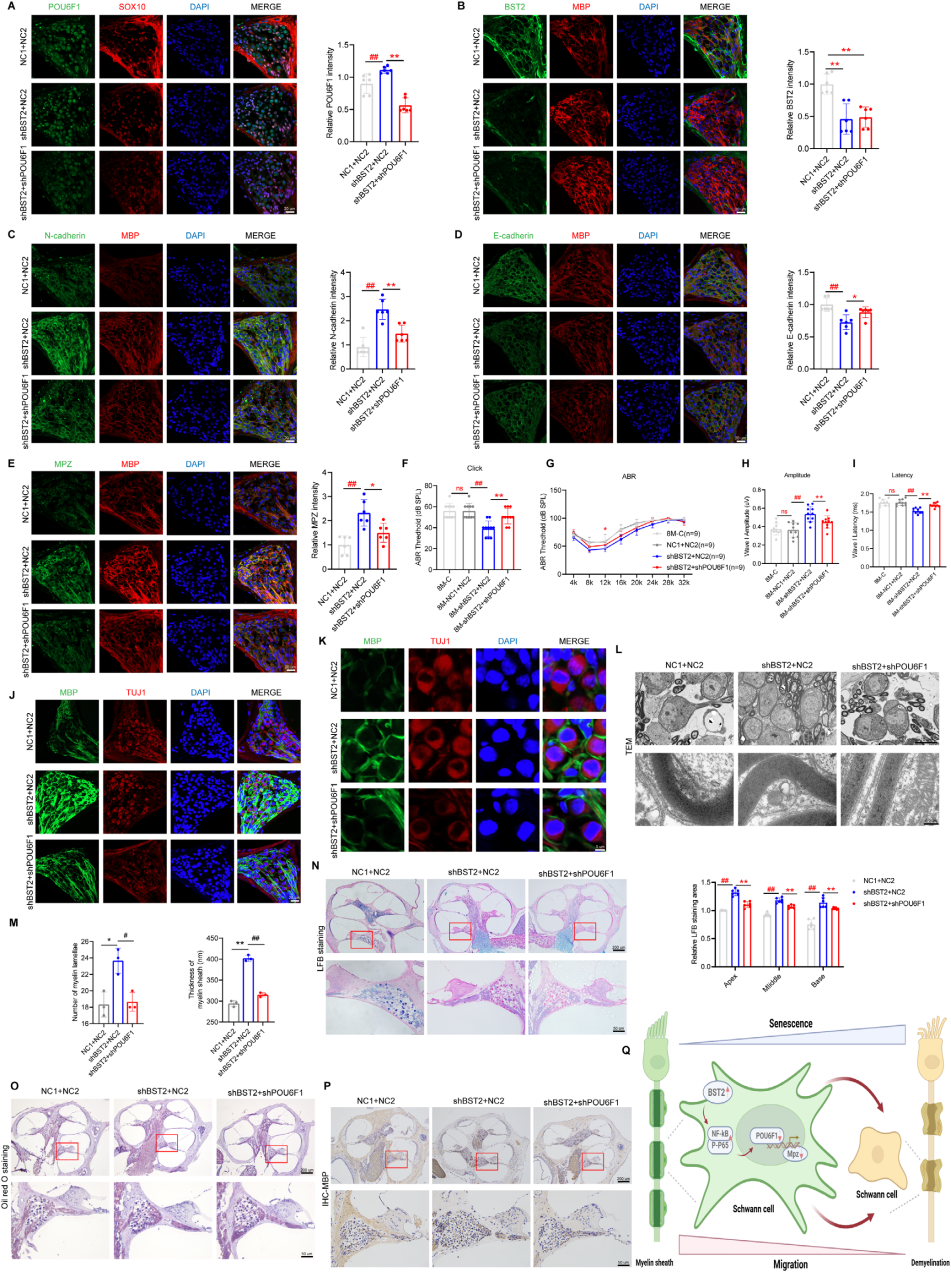
Downregulation of POU6F1 mediated by BST2 disrupts Schwann cell migration and promotes demyelination in ARHL. (A) Expression of POU6F1 (green) and SOX2 (red) in the SGNs of the cochlea in NC1 + NC2, shBST2 + NC2 and shBST2 + shPOU6F1. Immunofluorescence showed expression of BST2 (B), N‐cadherin (C), E‐cadherin (D), and MPZ (E) (green) in the cochlea of NC1 + NC2, shBST2 + NC2, and shBST2 + shPOU6F1. SCs were labeled with anti‐MBP (red). (F‐G) Click values and ABR thresholds to pure tones were measured in 8M‐C, NC1 + NC2, shBST2 + NC2, and shBST2 + shPOU6F1. (H, I) Latency and amplitude of ABR wave I were measured with click at SPL of 90 dB in 8M‐C, NC1 + NC2, shBST2 + NC2, and shBST2 + shPOU6F1. (J, K) Expression of MBP (green) in SGNs of NC1 + NC2, shBST2 + NC2, and shBST2 + shPOU6F1 by immunofluorescence. SGNs were labeled with anti‐TUJ1 (red). Nuclei were stained with DAPI (blue). (L) TEM images of the cochlea from NC1 + NC2, shBST2 + NC2, and shBST2 + shPOU6F1. (M) Quantitative analysis of myelin lamellae and myelin sheath thickness. (N) LFB staining showed the distribution of myelin in the cochlea of 8M‐C, 8M‐NC2, and 8M‐oePOU6F1. (O) Oil Red O staining showed the lipid distribution in the cochlea of NC1 + NC2, shBST2 + NC2, and shBST2 + shPOU6F1. (P) Immunohistochemistry was used for detecting MBP expression in the cochlea of NC1 + NC2, shBST2 + NC2, and shBST2 + shPOU6F1. (Q) The illustration indicates that increased BST2 expression in Schwann cells leads to cochlear nerve demyelination, accelerating hearing loss in ARHL. The upregulation of BST2 reduces POU6F1 expression by activating the NF‐κB pathway, which impairs MPZ transcription and Schwann cell migration, ultimately contributing to neural demyelination in ARHL. Data are presented as means ± SEM. *p < 0.05, **p < 0.01. ^#^ *p* < 0.05, ^##^ *p* < 0.01.

Dual‐virus semicircular canal injection revealed that POU6F1 knockdown partially reversed shBST2‐induced enhancement of cochlear SCs migration, concurrently attenuating N‐cadherin upregulation and partially blocking E‐cadherin downregulation (Figure 6A–D). Additionally, compared to the shBST2 + NC2 group, the double knockdown group exhibited further decreased MPZ expression, elevated click and pure‐tone auditory thresholds, prolonged I wave latency, and reduced amplitude (Figure 6E–I). The double‐knockdown disrupted myelin sheath integrity prematurely, as evidenced by a reduced proportion of SGNs with intact myelin sheaths, compromised myelin integrity, loose and disorganized residual myelin lamellae, with some regions showing lamellar fracture and separation, forming typical ‘onion skin‐like’ structures (Figure 6J–M). Furthermore, there was a reduced area of myelin staining in the cochlea (Figure 6N), along with a decrease in the expression of myelin sphingomyelin and myelin basic protein (Figure 6O,P). These findings suggest that BST2 reduces SCs' migratory function in ARHL by downregulating POU6F1, thereby contributing to demyelination.

## 4 Discussion

In the study, we demonstrate that BST2 expression increases in cochlear SCs during aging, correlating with diminished migratory capacity and progressive demyelination. Crucially, specific knockdown of BST2 in SCs could significantly restore SC migration, attenuate demyelination, and improve auditory function in aged mice, which reveals a novel molecular mechanism underlying auditory nerve dysfunction in ARHL, holding significant implications for understanding and treating cochlear nerve demyelination.

In the PNS, the myelination formed by SCs is essential for the efficient saltatory conduction of action potentials (APs) and the synchronized transmission of nerve impulses (Wan and Corfas 2017). Myelin sheath damage can lead to a variety of peripheral demyelinating disorders, including Charcot–Marie–Tooth disease (CMT) and Guillain‐Barré syndrome (GBS) (Gong et al. 2024; Pashkova et al. 2024). Notably, demyelination is associated with various forms of hearing loss, such as hidden hearing loss, noise‐induced deafness, drug‐induced ototoxicity, central deafness, and syndromic deafness (Kurioka et al. 2016; Wan and Corfas 2017; Guerrieri et al. 2023; Kedeshian et al. 2024). During demyelination, SC migration is widely recognized as a prerequisite for myelin maintenance and repair following acute injury, facilitating the formation of Büngner bands to guide axonal regeneration and remyelination (Guan et al. 2023; Wang et al. 2024). However, its role in age‐related cochlear degeneration remains incompletely understood. Previous studies have indicated that impaired SC migration in the aging cochlea disrupts the dynamic balance between demyelination and remyelination, leading to progressive myelin loss (Poleg et al. 2024). In recent years, C57BL/6 mice are widely employed as a model for demyelination research and are known to develop early‐onset hearing impairment due to the *Cdh23* mutation (Lu et al. 2024; Cheshmehsangi et al. 2025; Liu et al. 2025; Yin et al. 2025). However, in our study, the demyelinating features observed in the cochlea of 12‐month‐old mice corresponded with the time course of hearing deterioration in C57BL/6J mice, indicating that the associated neuropathological changes may not rely on the *Cdh23* mechanism. Notably, there are significant differences in hearing loss features between the C57BL/6J and C57BL/6N (Kendall and Schacht 2014; Mekada and Yoshiki 2021). We chose C57BL/6J as the animal model for this research because of its high genetic stability and its display of progressive cochlear aging traits akin to those in humans, such as the loss of hair cells from the cochlear base to apex, degeneration of SGNs, and stria vascularis deterioration (Bowl and Dawson 2015; Castano‐Gonzalez et al. 2024). By cellular and animal models, our findings further demonstrate that aged SCs exhibit reduced migratory capacity, characterized by decreased N‐cadherin and increased E‐cadherin expression. Additionally, we identify impaired SC migration as an early pathogenic event in ARHL that precedes overt demyelination. Restoration of migratory ability enables SCs to effectively translocate to axonal contact sites, thereby promoting myelin preservation and recovery of auditory function.

Within this pathogenic process, BST2 emerges with unexpected significance. Traditionally studied in immune regulation and antiviral responses, BST2 surfaces here as a key regulator of SCs migration in the aging cochlea (Shan et al. 2023; Liao et al. 2025). We identified BST2 as the most significantly upregulated gene in aged cochlear SCs, with validation at both mRNA and protein levels that correlated strongly with hearing threshold increases and demyelination severity. This sensory‐specific role meaningfully extends BST2's established functions: while its dysregulation exacerbates neuroinflammation in Alzheimer's disease and multiple sclerosis, we demonstrate SC‐autonomous regulation of migratory function (Manouchehri et al. 2021). Notably, in the aging cochlea, the upregulation of BST2 expression may be associated with the chronic activation of inflammatory factors such as interleukin‐6 (IL‐6) and tumor necrosis factor‐alpha (TNF‐α), as well as immune pathways, forming a vicious cycle that exacerbates glial cell dysfunction (Guerrieri et al. 2023; Lu et al. 2024). Meanwhile, studies have demonstrated that BST2 knockout can delay demyelination in experimental autoimmune encephalomyelitis models, further underscoring its widespread importance in the nervous system (Manouchehri et al. 2021; Li, Ye, et al. 2024; Li, Shao, et al. 2024). This suggests BST2 may represent a fundamental regulator of glial function in neural homeostasis.

This study builds upon established knowledge of BST2's involvement in migration regulation through ERK, NF‐κB, and AKT pathways (Li et al. 2023; Shan et al. 2023; Zheng et al. 2024). Among these pathways, NF‐κB serves as a master regulator of inflammatory responses and is known to modulate cell migration in various pathological conditions (Liao et al. 2025). This study demonstrates that elevated expression of BST2 in aged Schwann cells activates the NF‐κB signaling pathway. In addition, we reveal this activation directly suppresses the transcription factor POU6F1, which in turn regulates MPZ transcription. Previous studies have reported that POU6F1 is widely expressed in the nervous system (Cui and Bulleit 1998). Overexpression of POU6F1 in neurons promotes dendritic growth and synaptic development (McClard et al. 2018), while its expression is reduced following nerve injury. Wu et al. (2001) proposed that POU6F1, as a transcription factor, participates in myelin maintenance by regulating the MPZ promoter, a conclusion substantiated by our current findings. This glycoprotein constitutes > 50% of peripheral myelin protein and mediates homophilic adhesion critical for compact myelin formation (Han et al. 2025). MPZ deficiency causes severe demyelination in Charcot–Marie–Tooth disease (Christou et al. 2025). Our findings resonate with studies linking POU6F1 to peripheral nerve regeneration through JNK/c‐Jun signaling (Toda et al. 2008), yet diverge by revealing its novel role in maintaining myelin integrity during aging. The ChIP‐qPCR confirmed POU6F1 binding to the −217/−245 region of the MPZ promoter, while POU6F1 overexpression restored MPZ expression and rescued myelin integrity, firmly establishing this axis as central to ARHL pathogenesis. This regulatory pathway demonstrates that inflammation plays a crucial role in the pathogenic mechanism of ARHL (Lu et al. 2024), but uniquely positions BST2 as the orchestrator of inflammatory cascades specifically affecting glial function.

To resolve pathway hierarchy beyond correlative evidence, we used a dual‐viral intervention strategy in cochlea for the first time, co‐administering AAV9‐MBP‐shBST2 and AAV9‐MBP‐shPOU6F1 via semicircular canal injection. We achieved efficient co‐transduction without viral interference (Wang et al. 2025; Wu et al. 2025; Zong et al. 2025). The abolition of pro‐migratory benefits when shPOU6F1 accompanied BST2 inhibition definitively established POU6F1 as the principal downstream effector, resolving ambiguity about pathway specificity in the cochlear context.

The translational potential of targeting the BST2/POU6F1 axis is substantial. BST2 knockdown not only restored SCs migration but functionally reversed hearing loss, evidenced by 15–20 dB threshold improvements at critical speech frequencies (8–16 kHz) and wave I amplitude recovery. This positions BST2 inhibition as a viable strategy to preserve cochlear myelin integrity, potentially through repurposed immunomodulators or novel nanocarrier‐delivered siRNAs.

This study has certain limitations. The experiments were conducted exclusively in a single animal and cell model, which may not fully recapitulate the pathological changes in patients with ARHL. Additionally, the interactive role between BST2 and inflammation in the pathogenesis of ARHL has not been fully elucidated. In future research, we plan to incorporate multiple in vivo and in vitro models, as well as human samples, to further investigate the mechanistic roles of BST2 and inflammatory processes in ARHL and other forms of hearing loss. Meanwhile, we will also conduct preclinical translational studies to evaluate the potential therapeutic value of targeting BST2.

In conclusion, our research findings reveal that the upregulation of BST2 in SCs triggers the activation of NF‐κB and the suppression of POU6F1 in ARHL. Together, these effects impair the migration of SCs and the stability of myelin, ultimately resulting in demyelination. This finding provides a potential therapeutic target for ARHL.

## 5 Conclusion

This study identifies the BST2‐NF‐κB‐POU6F1 axis as a key driver of demyelination in ARHL. BST2 upregulation in aging Schwann cells impairs migration via NF‐κB‐mediated POU6F1 suppression, reducing MPZ expression and myelin integrity. Targeted BST2 knockdown restored SC migration, attenuated demyelination, and improved auditory function in ARHL mice. These findings position BST2 as a therapeutic target for preserving hearing in aging populations.

## Author Contributions

M.L. and B.W. designed the study. Q.L. and H.Y. performed experiments, conducted statistical analysis, and wrote the manuscript. H.C., J.Y., J.W., L.Z., and T.L. contributed to data curation, project administration, and resource supervision. All authors reviewed and approved the final manuscript.

## Funding

This work was supported by the S&T Program of Hebei (20577716D) and the Medical Talents cultivation program of the People's Government of Hebei Province (ZF2023114).

## Ethics Statement

The Ethics Committee of The Second Hospital of Hebei Medical University approved all treatments administered in this study (No. 2022‐AE308). We hereby confirm that this study has been reported in compliance with the ARRIVE guidelines.

## Conflicts of Interest

The authors declare no conflicts of interest.

## Supporting information


**Figure S1:** Expression of BST2 and POU6F1 in the RSC96. (A, B) The qRT‐PCR and western blot assays showed the siBST2 knockdown efficiency. (C, D) The qRT‐PCR and Western blot assays showed the oePOU6F1 overexpression efficiency. (E, F) The qRT‐PCR and western blot assays showed the siPOU6F1 knockdown efficiency. Data are presented as means ± SEM. *p < 0.05, **p < 0.01 versus NC.
**Table S1:** Sequences of primer, shRNA used in this study.

## Data Availability

The data that support the findings of this study are available on request from the corresponding author. The data are not publicly available due to privacy or ethical restrictions.
