# Supplementary material for: Downregulation of BST2 Rescues Cochlear Nerve Demyelination in Age‐Related Hearing Loss via Enhancing Schwann Cell Migration
Source: Aging Cell. 2025 Dec 14;25(1):e70325. doi: 10.1111/acel.70325 (PMC12741203; doi:10.1111/acel.70325)
Supplement: Supplementary file 1 — Figure S1: Expression of BST2 and POU6F1 in the RSC96. (A, B) The qRT‐PCR and western blot assays showed the siBST2 knockdown efficiency. (C, D) The qRT‐PCR and Western blot assays showed the oePOU6F1 overexpression efficiency. (E, F) The qRT‐PCR and western blot assays showed the siPOU6F1 knockdown efficiency. Data are presented as means ± SEM. *p < 0.05, **p < 0.01 versus NC. Table S1: Sequences of primer, shRNA used in this study. [file ACEL-25-e70325-s001.docx]

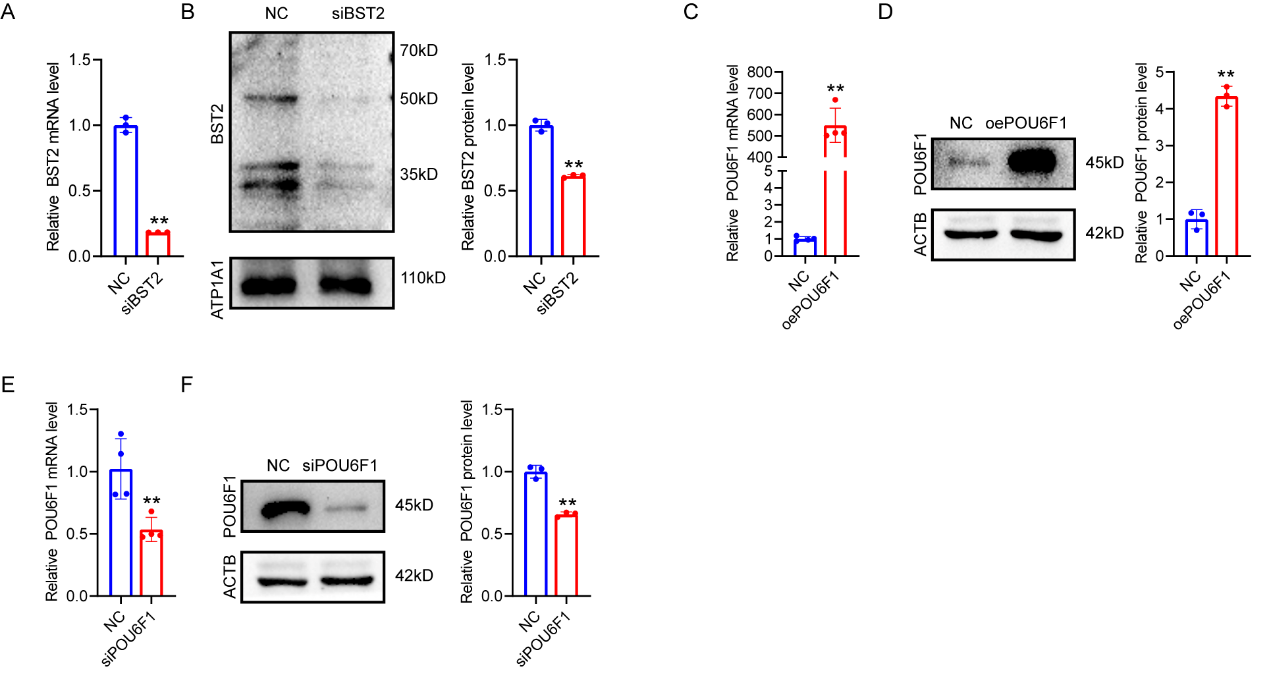


Figure S1. Expression of BST2 and POU6F1 in the RSC96. (A-B) The qRT-PCR and western blot assays showed the siBST2 knockdown efficiency. (C-D) The qRT-PCR and western blot assays showed the oePOU6F1 overexpression efficiency. (E-F) The qRT-PCR and western blot assays showed the siPOU6F1 knockdown efficiency. Data are presented as means ± SEM. ** P* < 0.05, *** P* < 0.01 vs NC.

**Table S1. Sequences of primer, shRNA used in this study**

| Bst2-F | Mouse | GACCCAATAGTTGGCAGGTCA |
| --- | --- | --- |
| Bst2-R | Mouse | CAGGAGATGCAATCTGGGCT |
| Bst2-F | Rat | ACCCAATAGTTGGCAGGTCAC |
| Bst2-R | Rat | GCAATCTGGGCTCCACCTAA |
| siBst2-F | Rat | CAAAAGGAAAUUUCUACCATT |
| siBst2-R | Rat | UGGUAGAAAUUUCCUUUUGTT |
| shBst2-F | Mouse | CACAGUUGUUUGAAGUCACUATT |
| shBst2-R | Mouse | UAGUGACUUCAAACAACUGUGTT |
| Cdh5-F | Rat | TGAAGAACGAGGACAGCAACT |
| Cdh5-R | Rat | GAGATGAGCACAGGCAGGTA |
| Hoxb8-F | Rat | AGACCTACAGCCGCTACCA |
| Hoxb8-R | Rat | TCCACTTCATTCTCCGATTCTG |
| Sdhaf1-F | Rat | GCAAGTTCTGAGCCTGTACC |
| Sdhaf1-R | Rat | ATCACTATCGTCCAGCCTGTT |
| Lqcc-F | Rat | AGCCTACTAAGATGTGCCTGAA |
| Lqcc-R | Rat | ACCAGAGTTCCGATAGAGTTCC |
| Gchfr-F | Rat | TCATCAGCACTCAGATCCGTAT |
| Gchfr-R | Rat | TTCCAGCTTGTCCAGGACTATT |
| Mip-F | Rat | ATGACGAGAGGCGGAATGG |
| Mip-R | Rat | GGTGAGGATAGCAGGAGCAA |
| Oas1a-F | Rat | GCTGGACAAGTTCATAGAGGTT |
| Oas1a-R | Rat | GACAGTATCTCGGAAGCATCTC |
| Pou6f1-F | Rat | GCCAGCCTACAGCCAATCA |
| Pou6f1-R | Rat | TCTGCCTCGTTCAACCACTT |
| Si-Pou6f1-F | Rat | GUCAGUCAGUUGGUAUCAATT |
| Si-Pou6f1-R | Rat | UUGAUACCAACUGACUGACTT |
| Sh-Pou6f1-F | Mouse | GUCAGUCAGUUGGUAUCAATT |
| Sh-Pou6f1-R | Mouse | UUGAUACCAACUGACUGACTT |
| Mpz-F | Rat | GTTGCTGCTGTTGCTCTTCTA |
| Mpz-R | Rat | GTTCTTGAGGCTGGTTCTACTG |
| ChIP 1-F | Rat | AGCAGAAACTCAGACGTCA |
| ChIP 1-R | Rat | CTGAGAGTGGTAGTGTATGCC |
| ChIP 2-F | Rat | CGAGCCAAGCTCTTTATC |
| ChIP 2-R | Rat | GTATCCAGTGTCCTGCCTC |
| ChIP 3-F | Rat | GAGGCAGGACACTGGATAC |
| ChIP 3-R | Rat | GAGTGAACCCAGAAGAACAGC |
